# Improvement of power quality parameters using modulated-unified power quality conditioner and switched-inductor boost converter by the optimization techniques for a hybrid AC/DC microgrid

**DOI:** 10.1038/s41598-022-26001-8

**Published:** 2022-12-15

**Authors:** Nima Khosravi, Abdollah Abdolvand, Adel Oubelaid, Yawer Abbas Khan, Mohit Bajaj, Scott Govender

**Affiliations:** 1 Department of Electrical and Instrumentation Engineering, R&D Management of NPC, Tehran, Iran; 2 Department of Engineering, Islamic Azad University, Aligoudarz Branch, Aligoudarz, Iran; 3 Laboratoire de Technologie Industrielle et de l’Information, Faculté de Technologie, Université de Bejaia, 06000 Bejaia, Algeria; 4 Department of Electrical and Electronics Engineering, BIET, Hyderabad, India; 5 Department of Electrical Engineering, Graphic Era (Deemed to be University), Dehradun, 248002 India; 6 Department of Power Systems Operation and Planning, Power Electrical Industry Consultants Co, Limbe, Malawi

**Keywords:** Energy grids and networks, Power distribution

## Abstract

This study aims to improve the quality of operation parameters of the stand-alone hybrid microgrids (HMGs). The proposed module for the AC microgrid (ACMG) is a modulated-unified power quality conditioner (M-UPQC). Furthermore, the suggested component for the DC microgrid (DCMG) is a switched-inductor boost converter module (S-IBCM). The M-UPQC control method is based on inverter modules and the system resonant features. The aim of S-IBCM applied is to improve DC microgrid (DCMG) efficiency. In this paper, the research challenge consists of two sections: first, adjusting the control parameters of M-UPQC by the black hole optimization (BHO), Harris hawk optimization (HHO), and grasshopper optimization algorithm (GOA) techniques, respectively; second, presenting a new design of the BC module called S-IBCM to increase DCMG efficiency. The programmed multi-objective functions (MOFs) for M-UPQC contain the harmonic parameters. Finally, according to output results, the performance conditions for ACMG and DCMG divisions achieve significantly improved by the proposed modules adopted. Furthermore, the performance of the M-UPQC operating under static and dynamic disturbances is tested through an experimental setup.

## Introduction

MGs are units that can provide electric energy locally. The proper efficiency of these units has led to the increasing development of these platforms over the world. The nature of MGs consists of distributed energy resources (DERs). The role of distributed energy resources (DERs) in decreasing environmental contaminants is irrefutable, where these sources are extensively employed for energy production in MGs^1–3^. On the other hand, one of the most matters is the power quality (PQ) of power grids, which is the topic of discussion in articles^4–7^. A stability appraisal and execution investigation of the new controller for PQ conditioning in MGs are presented in Ref.^8^. In mentioned paper has presented a novel controller for UPQC connected MG, which controls the switching of voltage source converters (VSCs) of UPQC by carrying the derived error at three various adjustments and then incorporating it over time for enhancing and reinforcing PQ issues. The presented results indicate the acceptable performance of the mentioned controller. Reactionary management of HMG with harmonic unstable loads underneath outages and faults, investigated in Ref.^9^. The defined goals in the mentioned paper are to decrease the harmonics, the unstable load managment, deal with the outage of resources and short-circuits, supply backup techniques, and feed required loads underneath all occurrences. A review of control procedures of parallel-interfaced voltage source inverters (VSIs) for distributed power generation systems is discussed in Ref.^10^. The mentioned paper includes various control structures to be examined separately. In Ref.^11^, a coordinate harmonic suppression method of a DCMG has been proposed. The mentioned method is proposed for two goals. First, quench the voltage pulsing in the DC bus capacitor, which seeks to mitigate the negative effect of the grid voltage harmonics. Furthermore, to obtain sinusoidal outcome voltage, that is, to improve the PQ of the MG by correctly eliminating the harmonics raised by the non-linear loads. Two issues of overshoot and undershoot concerns in DC-link voltage in a DER grid have been investigated in Ref.^12^. In the mentioned paper, the system frequency management in dynamics and steady-state cases and compensation of power among different energy sources and a battery energy storage system (BESS), is achieved utilizing the sliding mode control (SMC). The output results of the mentioned article show the performance effect of the proposed method (SMC) in achieving the objectives of the problem. In the literature^13–16^, the harmonic analysis topic of HMG networks with the presence of filter modules has been comprehensively presented. Based on the cases presented in the above literature, filter modules can be considered one of the best solutions to reduce the range of harmonic components of MGs and power systems. Some disadvantages of the above-cited papers in comparison with the scheme presented in this paper are as follows:

- The suggested approach is on time to execute, in comparison with time limitation concerns in enforcing metaheuristic methods^13–16^.
- Contrary to the literature presented in Refs.^8,9,11,13^, which has a relatively complex control structure, the proposed approach in this paper is not complicated.
- The techniques suggested in the papers^3,12^ are more effective for systems with generators and similar machines.

After examining the literature related to the harmonic and fluctuating cases; following, the authors will introduce some literature that exclusively addressed the proposed module (UPQC). The UPQC is the combination of series and parallel inverters, tied back-to-back on the dc side, with a share to a common DC capacitor. The UPQC series element is accountable for the mitigation of the supply side disruptions: voltage sags/swells, flicker, voltage unbalance, and harmonics. It inserts voltages to keep the load voltages at the expected grade; balanced and distortion-free. The parallel element is accountable for reducing the current quality troubles generated by the consumer: insufficient power factor, load harmonic currents, load imbalance, etc. It infiltrates currents in the AC system such that the source currents evolve balanced sinusoids and in phase with the source voltages. The general function of UPQC largely relies on the series and parallel inverters controller^17–22^.

In Refs.^23,24^ proposes the designing procedure for open UPQC integrated with PV in radial distribution grids to enhance the power efficiency. The suggested strategy is contained in the forward–backward sweep load flow to select the operating components, such as busbar voltage. In the mentioned paper, the ‘OF’ includes the acquisition and operating expenses of inverters, battery and PV collection. An optimization algorithm has been applied in the above study. However, the suggested algorithm may not be effective enough for online execution. The novel control approach for UPQC to improve DC-link voltage ripple and PQ enhancement is suggested in Refs.^25,26^. Output results in two modes of simulation and experimental indicate the proper performance of UPQC by applying the control strategies proposed in this literature. In this paper, the HMG function issue is analyzed based on two compensator modules (M-UPQC and S-IBCM). In the following, the HMG operation has improved by applying the mentioned modules. Therefore, the main destinations and contributions of the paper are the following:

- The provided DCMG terminal with a BCM to reinforce the output components (V and I) of the mentioned terminal.
- A switched inductor is embedded in the BCM structure to define paths to reduce the basic parameters fluctuation range of the grid.
- The regulation of the M-UPQC controller features using the optimization approach. The proposed method is a consideration in the multi-objective functions (MOFs) formation. The programmed MOFs include two segments and contain the voltage harmonic loop and the current harmonic loop. Each of the designed MOFs will be optimized based on the BHO technique to adjust the PI-MR control components to explore and find the most undersized amplitude of harmonic fluctuations caused by the grid. In case of varying working conditions, the controller parameters will be self-tuned depending on the new conditions. All the above will be possible by maintaining and observing the designed constraints.
- The HHO and GOA techniques will apply as complementary approaches for verification and comparison with the results acquired from the BHO technique.

The rest of the paper is organized as follows: In “The case study structure” section of this paper, the configuration topology of the case study is presented. In “M-UPQC system” section of this paper, the configuration topology of the proposed M-UPQC is presented. In “The proposed MOFs” section presents the proposed MOFs to solve the case study problems. The research results are discussed in “Results and discussion” section. The conclusions of this paper are presented in “Conclusion” section.

## The case study structure

**Figure 1:**
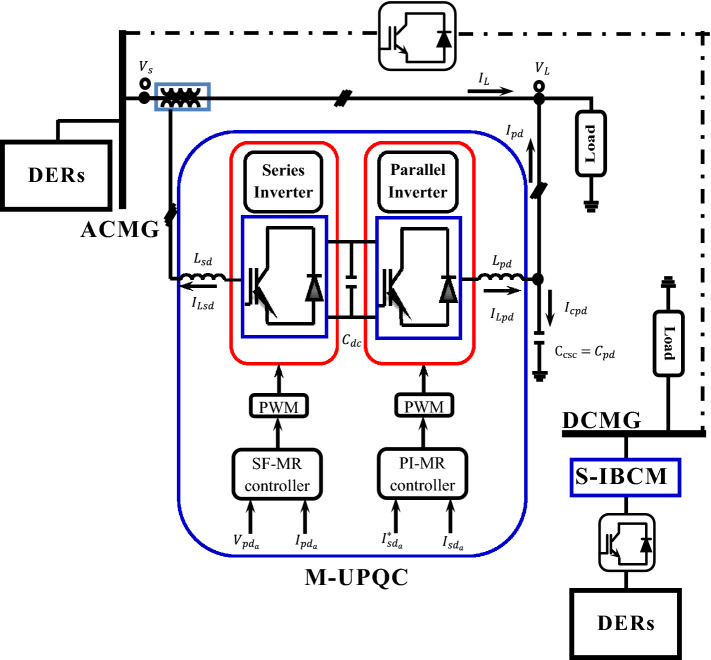
The structure of the HMG.

The proposed HMG is composed of two separate parts. The DERs of the ACMG grid involve wind farms and microturbine generators (MTGs). The power generation structure for the DCMG grid includes PV cells and a BESS that will be connected to the mentioned grid by the S-IBCM interface element. The specification of each component employed in the studied system will be presented in the appendix. The proposed design of the HMG is shown in Fig. 1.

### BESS configuration

The BESS application is to serve the voltage and frequency stability in uncertainty states^27–29^. The battery type applied in the BESS is a generic configuration, and the voltage source is controllable in the suggested model. According to the state of charge (SOC), the no-load voltage (${E}_{NL}$) will be calculated from relation (1). Furthermore, to increase the BESS efficiency, the S-IBCM system will be matched as an interface system.

1 $${E}_{NL}={E}_{BCV}-K\frac{1}{SOC}+ {C}^{-DQ\left(1-SOC\right)},$$

where ${\mathrm{E}}_{\mathrm{BCV}}$ is the stationary voltage of battery (*V*), *K* is the polarization voltage (*V*), *Q* is the battery capacity (*Ah*), *C* and *D* indicators are the battery characteristic for charge and discharge mode.

### PV configuration

**Figure 2:**
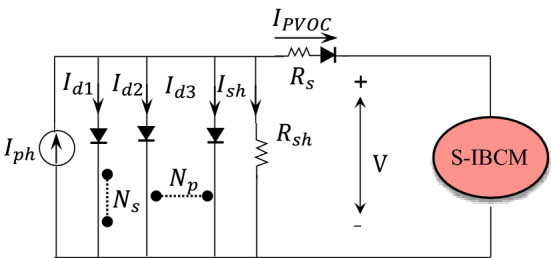
The equivalent circuit of the PV system.

The PV equivalent circuit is shown in Fig. 2. This module includes the series (${N}_{s}$) and parallel (${N}_{p}$) solar cells. Comparable to BESS, the PV output will be connected to the S-IBCM to increase the system's efficiency. According to the equivalent circuit of the module, the PV equations will be formulated as follows:

2 $${\rm A}_{x}= \left[\frac{q(V+ {N}_{s} {R}_{s}{ I}_{PVOC}/{N}_{p})}{n k{ N}_{s}T}\right],$$

3 $${\rm A}_{y}= \left[\frac{V+{N}_{s}{ R}_{s }{ I}_{PVOC}/{N}_{p}}{{R}_{sh}{N}_{s}/{N}_{p}}\right],$$

4 $${\rm B}_{x}= \left[\frac{q(V+ {N}_{s} {R}_{s}{ I}_{PVOC}/{N}_{p})}{{n}_{1 }k {N}_{s}T}\right],$$

5 $${B}_{y}= \left[\frac{q(V+ {N}_{s} {R}_{s}{ I}_{PVOC}/{N}_{p})}{{n}_{2 }k {N}_{s}T}\right],$$

6 $${I}_{PVOC}={I}_{ph}{N}_{p-}{I}_{dx}{N}_{p}({e}^{\mathrm{A}x}-1){-\mathrm{A}}_{y},$$

7 $${I}_{PVOC}={I}_{ph}{N}_{p-}{I}_{d1}({e}^{{\mathrm{B}}_{x}}-1)-{I}_{d2} -({e}^{{\mathrm{B}}_{y}} -1)-{\mathrm{A}}_{y},$$

where *e* is the electron charge (1.602 $\times {10}^{-19}C$), PV cell output voltage (*V*), ${R}_{s}$ is ohmic resistance (Ω), ${I}_{PVOC}$ is PV output current (A), ‘n’ is branch type, *K* is the Boltzmann constant value (1.38 $\times {10}^{-23}\frac{j}{k}$), ${T}_{c}$ is the reference temperature (25c).

### S-IBCM interface

**Figure 3:**
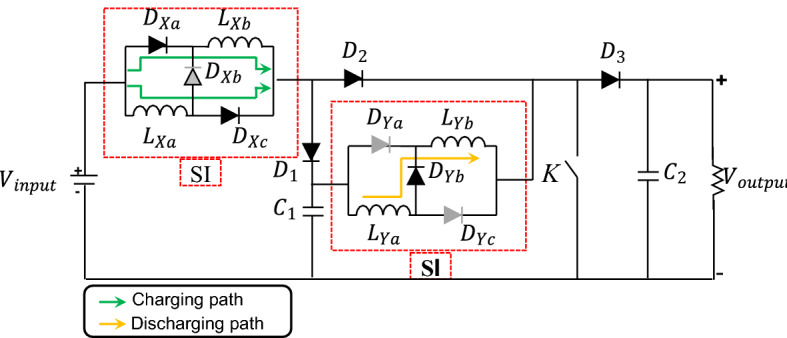
The structure of the S-IBCM.

**Figure 4:**
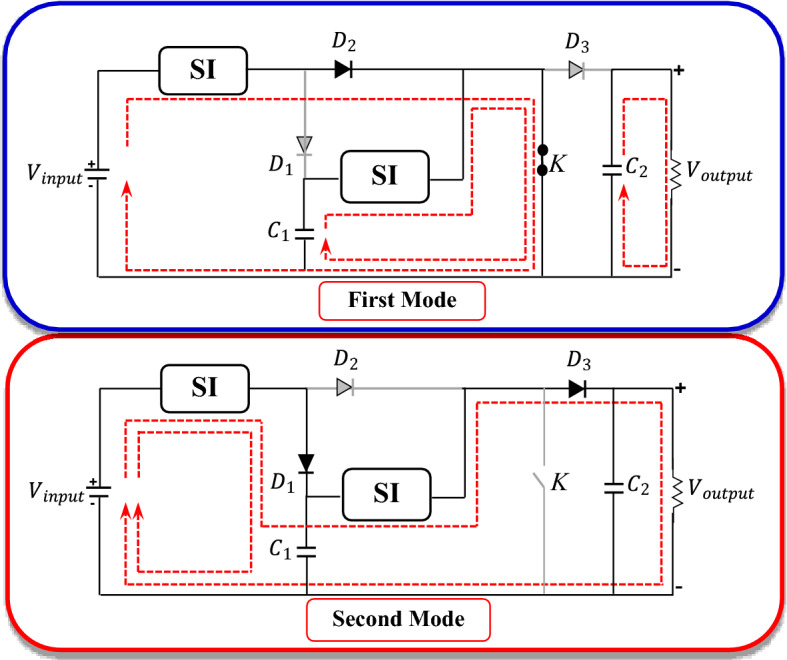
The SIBCM operation modes.

The S-IBCM is the interface module between three components containing PV, BESS, and the DC-link block that following is connected to the DCMG section. The mentioned equipment can have various operating modes, depending on the control and type of operation^30,31^. The structure of the S-IBCM is shown in Fig. 3. This equipment consists of two switched inductor (SI) components. The SI charging and discharging path is shown in Fig. 3 in different colors. The S-IBCM consists of two operating modes, shown in Fig. 4. The first mode is 0-D ${T}_{s}$, and the second mode is D ${T}_{s}$− ${T}_{s}$.

where D is duty ratio and ${T}_{s}$ is switching frequency. With closing the *K* key (turned on), the voltage will be obtained from the following equations:

8 $${V}_{LXa}={V}_{LXb}= {V}_{input}-4{V}_{d},$$

9 $${V}_{LYa}={V}_{LYb}= {V}_{C1}-3{V}_{d}.$$

For off state:

10 $${V}_{LX}= \frac{{V}_{input}-{V}_{c1}-4{V}_{d}}{2},$$

11 $${V}_{LY}= \frac{{V}_{C1}-{V}_{0}-4{V}_{d}}{2}.$$

For $${L}_{X}$$ component:

12 $$\left({V}_{input}- 4{V}_{d}\right)D+\left(\frac{{V}_{input}-{V}_{c1}-4{V}_{d}}{2}\right)(1-D) = 0,$$

13 $${V}_{C1}=\left(\frac{1+D}{1-D}\right){V}_{input}-\left(\frac{1+D}{1-D}\right)4{V}_{d}.$$

For $${L}_{Y}$$ component:

14 $$\left({V}_{C1}- 3{V}_{d}\right)D+\left(\frac{{V}_{C1}-{V}_{0}-4{V}_{d}}{2}\right)(1-D) = 0,$$

15 $${V}_{0}=\left(\frac{1+D}{1-D}\right){V}_{C1}-\left(\frac{2+D}{1-D}\right)2{V}_{d}.$$

For Eq. (12 and 13):

16 $${V}_{0}={\left(\frac{1+D}{1-D}\right)}^{2}{V}_{input}- 2\left(\frac{{D}^{2}+3D+4}{{\left(1-D\right)}^{2}}\right){V}_{d}.$$

Assuming ignoring the internal voltage droop, the voltage conversion rate relationship is as follows:

17 $${V}_{0}= {\left(\frac{1+D}{1-D}\right)}^{2}{V}_{input}.$$

### M-UPQC system

Here, the proposed module for the grid AC part is M-UPQC. The mentioned module is the conventional version that has been modified in terms of control. The suggested modifications will be practical and effective in the functionality of the desired module. Furthermore, the presented M-UPQC classification is dual-inverted^32–34^. Because the preferred inverter feature is multi-level, and the neutral point clamped (NPC) module has been applied to improve system efficiency.

Here, the main challenge is the control of inverters. In the established classification, the control parameters (V and I) are non-sinusoidal, and the converters are managed according to the essential components of the mains voltages positive sequence to counterbalance and sinusoidal the (V and I) parameters. Based on the current source converter (CSC), load currents ($${{I}_{Lb}, I}_{La}$$ and $${I}_{Lc})$$ will be converted to the reference form (*d-q*) in the standard synchronous reference mode (SRM). The converted current $${I}_{Ld}$$ passes through the low pass filter (LPF) element and is displayed with an index ($${I}_{{Ld}_{dc}}$$). Finally, the general current is a combination of $${I}_{dc}$$ and $${I}_{{Ld}_{dc}}$$ represented by the index ($${I}_{{sd}_{d}}^{*}$$). Each of these current parameters will be obtained as:

18 $${I}_{Ld}=\sqrt{\frac{2}{3}} \left[\begin{array}{ccc}\mathrm{cos\theta }& -\frac{1}{2}\mathrm{cos\theta }+\frac{\sqrt{3}}{2}\mathrm{sin\theta }& -\frac{1}{2}\mathrm{cos\theta }+\frac{\sqrt{3}}{2}\mathrm{sin\theta }\end{array}\right]\left[\begin{array}{c}{I}_{La}\\ {I}_{Lb}\\ {I}_{Lc}\end{array}\right],$$

19 $${I}_{{sd}_{d}}^{*}={I}_{{Ld}_{dc}}+{I}_{dc},$$

where $\mathrm{sin\theta }$ and $\mathrm{cos\theta }$ are the rotating unit vector coordinates. The current parameters of the grid side will be obtained from the following relations:

20 $$\left[\begin{array}{c}{I}_{{sd}_{a}}^{*}\\ {I}_{{sd}_{b}}^{*}\\ {I}_{{sd}_{c}}^{*}\end{array}\right]=\sqrt{\frac{2}{3}} \left[\begin{array}{cc}1& 0\\ -\frac{1}{2}& \frac{\sqrt{3}}{2}\\ -\frac{1}{2}& -\frac{\sqrt{3}}{2}\end{array}\right]\left[\begin{array}{c}{I}_{{sd}_{d}}^{*}\mathrm{cos\theta }\\ {I}_{{sd}_{d}}^{*}\mathrm{sin\theta }\end{array}\right]=\left[\begin{array}{c}{I}_{{Sa}_{1}}^{*}\\ {I}_{{Sb}_{1}}^{*}\\ {I}_{{Sc}_{1}}^{*}\end{array}\right],$$

where ${I}_{{Sa}_{1}}^{*}, {I}_{{Sb}_{1}}^{*}$ and ${I}_{{Sc}_{1}}^{*}$ are the positive sequence parameter (PSP) currents. For the VSC component also, the output voltage parameter of the converter will also be controlled in phase along with the basic PSPs of the mains voltage. The reference mode (*abc*) voltage is as follows:

21 $$\left[\begin{array}{c}{V}_{La}^{*}\\ {V}_{Lb}^{*}\\ {V}_{Lc}^{*}\end{array}\right]=\left[\begin{array}{c}{V}_{{Pd}_{a}}^{*}\\ {V}_{{Pd}_{b}}^{*}\\ {V}_{{Pd}_{c}}^{*}\end{array}\right]=\left[\begin{array}{c}{V}_{Peak}^{*}\mathrm{sin\theta }\\ {V}_{Peak}^{*}\mathrm{sin}\left(\uptheta -{120}^{^\circ }\right)\\ {V}_{Peak}^{*}\mathrm{sin}\left(\uptheta -{240}^{^\circ }\right)\end{array}\right],$$

where ${V}_{La}^{*}$, ${V}_{Lb}^{*},$ and ${V}_{Lc}^{*}$ are the voltage references of the load voltages/parallel converter in the ‘abc’ stationary reference frame, ${V}_{Peak}^{*}$ is the maximum amplitude of the three-phase load voltages. The presented control scheme for M-UPQC exists as a hybrid design and consists of current source inverter (CSI) and voltage source inverter (VSI) modes. The CSI is produced accord joining the PI-MR with the CSC segment. The VSI is made by state feedback-multi resonant (SF-MR) and VSC components. The mentioned control modes will be defined as follows.

### CSI controller

**Figure 5:**
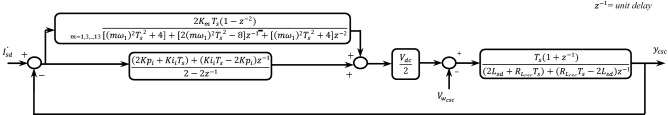
The CSI controller block diagram.

The management association between the CSC and PI-MR is shown in Fig. 5. The state space relation for the CSC segment will be defined as follows:

22 $$\underset{\dot{{x}_{csc}}}{{\left[\dot{{I}_{sd}}\right]}}=\underset{{A}_{csc}}{{\left[-\frac{{R}_{{L}_{csc}}}{{L}_{sd}}\right]}}\underset{{x}_{csc}}{{\left[{I}_{sd}\right]}}+\underset{{B}_{csc}}{{\left[\frac{{V}_{dc}}{{2L}_{sd}}\right]}}\left[{d}_{csc}\right]+\underset{{B}_{{w}_{csc}}}{{\left[-\frac{1}{{L}_{sd}}\right]}}\underset{{w}_{csc}}{{\left[{V}_{{w}_{csc}}\right]}},$$

23 $${y}_{csc}=\underset{{C}_{csc}}{{\left[1\right]}}\underset{{x}_{csc}}{{\left[{I}_{sd}\right]}},$$

where ${R}_{{L}_{csc}}$ is internal resistance, ${V}_{{w}_{csc}}$ is the voltage across the series transformer, ${d}_{csc}$ is the CSC duty cycle. According to the relations (22) and (23), the open loop transfer function (TF) of the CSC component is as follows:

24 $${G}_{csc}\left(s\right)=\frac{{I}_{sd}(s)}{{d}_{csc}(s)}=\frac{{V}_{dc}}{2}\frac{1}{{L}_{sd} s+{R}_{{L}_{sd}}}.$$

Given that the integrated PI-MR is a combination of a ‘P’–‘I’ parameters and a resonant condition, so TF of PI-MR can be defined as the following equation:

25 $${G}_{PI-MR}\left(s\right)=\frac{{Kp}_{i} s+{Ki}_{i} }{s}+\sum_{m=1}^{n}\frac{{K}_{m }s}{{S}^{2}+{\left({m\omega }_{1}\right)}^{2}},$$

where ${Kp}_{i} , {Ki}_{i}$ represents the P and I gains of the current PI-MR controller, respectively; ${K}_{m}$ is the resonant gain at a particular resonant frequency (${\omega }_{0}$ = ${m\omega }_{1}$); ${\omega }_{1}$ is the basic utility frequency; and *m* = 1, 3, …, 13 are the chosen resonant periods.

### VSI controller

**Figure 6:**
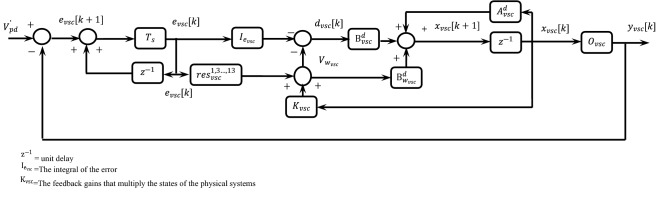
The VSI controller block diagram.

The management association between the VSC and SF-MR is shown in Fig. 6. The state space relation for the VSC segment will be defined as follows:

26 $$\underset{\dot{{x}_{vsc}}}{{\left[\begin{array}{c}\dot{{{I}_{L}}_{pd}}\\ \dot{{V}_{pd}}\end{array}\right]}}=\underset{{A}_{vsc}}{{\left[\begin{array}{cc}-\frac{{R}_{{L}_{vsc}}}{{L}_{sd}}& -\frac{1}{{L}_{vsc}}\\ \frac{1}{{C}_{vsc}}& 0\end{array}\right]}}\underset{{x}_{vsc}}{{\left[\begin{array}{c}{I}_{{L}_{pd}}\\ {V}_{pd}\end{array}\right]}}+\underset{{B}_{vsc}}{{\left[\begin{array}{c}\frac{{V}_{dc}}{{2L}_{pd}}\\ 0\end{array}\right]}}\left[{d}_{vsc}\right]+\underset{{B}_{{w}_{vsc}}}{{\left[\begin{array}{c}0\\ \frac{1}{{C}_{vsc}}\end{array}\right]}\underset{{w}_{vsc}}{{\left[{I}_{{w}_{vsc}}\right]}}},$$

27 $${y}_{vsc}=\underset{{O}_{vsc}}{{\left[\begin{array}{cc}0& 1\end{array}\right]}}\underset{{x}_{vsc}}{{\left[\begin{array}{c}{I}_{{L}_{pd}}\\ {V}_{vsc}\end{array}\right]}}.$$

where, ${R}_{{L}_{vsc}}$ is internal resistance,${I}_{{w}_{vsc}}$ is the current of the parallel converter, and ${d}_{vsc}$ is the duty-cycle of the VSC. For the SF-MR control component, considering the ${e}_{vsc}$ error integral in the control loop and also considering the resonant conditions (${1}^{th},{3}^{th},\dots ,{13}^{th}$), the state space matrix will be formulated as follows:

28 $$\underset{{\dot{x}}_{res}}{{\left[\begin{array}{c}\dot{{x}_{{res}_{{1}^{th}}}}\\ \dot{{x}_{{res}_{{3}^{th}}}}\end{array}\right]}}=\underset{{A}_{res}}{{\left[\begin{array}{cc}0& -{(n{\omega }_{1})}^{2}\\ 1& 0\end{array}\right]}}\underset{{x}_{res}}{{\left[\begin{array}{c}{x}_{{res}_{{1}^{th}}}\\ {x}_{{res}_{{3}^{th}}}\end{array}\right]}}+\underset{{B}_{res}}{{\left[\begin{array}{c}1\\ 0\end{array}\right]}}\left[{e}_{vsc}\right],$$

where '*n*' is defined based on the resonant term (${1}^{th},{3}^{th},\dots ,{13}^{th}$).

## The proposed MOFs

Another contribution of this research is the set of PI-MR control parameters by an approach based on controller gain coefficient optimization. The suggested strategy is defined in a MOF formation. The programmed OFs are harmonic loop based. The MOFs developed will be revamped by the BHO and HHO, and GOA techniques, respectively, to set the PI-MR control components to explore and discover the lowest harmonic oscillations' amplitude. The proposed method roadmap will be presented in the optimization algorithms flowcharts. Furthermore, after finishing the analysis stage and considering the problem restrictions, the responses will be re-checked. The preferred phases will continue until the most suitable retort is reached.

### BHO method

In this method, the concept of sucking stars by black hole (BH) is applied as a search space^35^. The initial population is created by stars and the value of each solution is examined. According to the following equation, the new position of each star is determined according to the previous star and the position of the BH.

29 $${x}_{i}^{new}={x}_{i}^{old}+SN\left({x}_{BH}-{x}_{i}^{new}\right), i= 1, 2\dots ,\mathrm{ n},$$

where ${x}_{i}$ represents the position of the star in the old and new positions, *SN* is a random number between [0, 1], n is the number of stars (solutions), and ${x}_{BH}$ is the position of the BH. After the star moves to the new position, it’s value function and the BH value function will be calculated. The radius of the event horizon of the BH will be obtained through the following relation:

30 $$REH=\frac{{f}_{BH}}{\sum_{i=1}^{N}{f}_{i}},$$

where ${f}_{BH}$ is the fitness value of the black hole and ${f}_{i}$ is the fitness value of the ${i}^{th}$ star. The implementation steps of the algorithm are as follows:

i. Initialize a population of stars with random locations in the search space *p*(*t*)$=\left\{{x}_{1},{x}_{2},..,{x}_{n}\right\}$. Randomly generated population of candidate solutions (the stars) are placed in the search space of some problem or function.
  Loop
ii. For each ${i}^{th}$ star, evaluate the OF.
  $${f}_{i}=\sum_{i=1}^{pop\_size}eval p\left(t\right), {f}_{BH}=\sum_{i=1}^{pop\_size}eval p\left(t\right).$$
iii. Select the best star that has the best fitness value as the black hole.
iv. Change the location of each star according to Eq. (29).
v. If a star reaches a location with lower cost than the black hole, exchange their locations.
vi. If a star crosses the event horizon of the black hole.
vii. Calculate the Eq. (30).
viii. When the distance between a candidate solution and the black hole (best candidate) is less than R, that candidate is collapsed and a new candidate is created and distributed randomly in the search space.
ix. Replace it with a new star in a random location in the search space.
x. Else, break
xi. If a termination criterion (a maximum number of iterations or a sufficiently good fitness) is met exit the loop.

**Figure 7:**
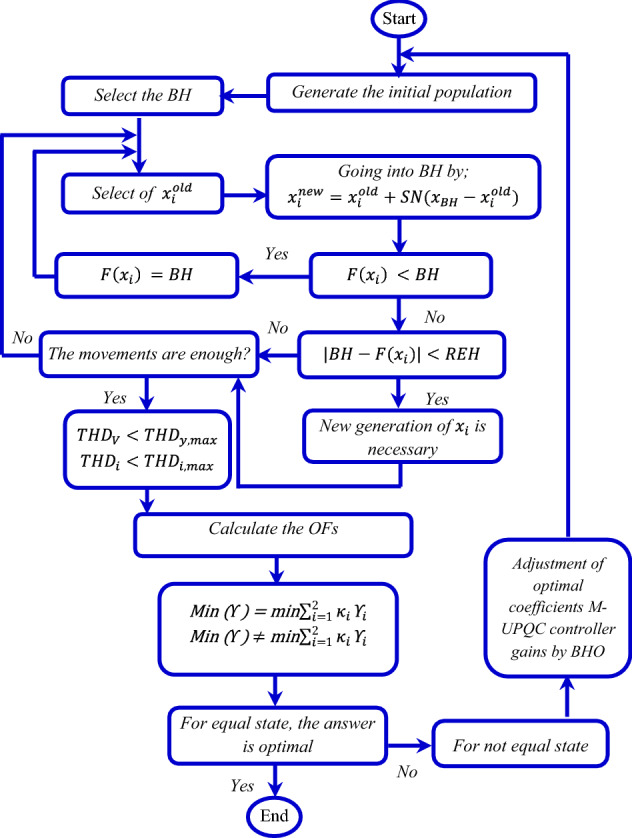
Flowchart of the proposed technique based on BHO method.

How to optimize the coefficients of each of the M-UPQC control components using the BHO method is presented in the roadmap of the case study in Fig. 7. Following, selected parameters of BHO are recorded in Table 1.

**Table 1:** Selected parameters of BHO algorithm.

Parameter	Value
Number of partials	20
Coefficient for the perturbation operator	20%
Number of groups	10
Iteration number	50

### HHO method

The principal concept of the HHO approach is the participatory manners and a pursuit technique of a Harris hawk in nature, which is understood as a shock attack. The strategy is formed from the two major sections of the exploration and exploitation stages. The siege process consists of two phases, including the soft-siege (SS) and hard-siege (HS)^36,37^. The soft-siege relations are as follows:

31 $${H}_{t}+1= \left\{\begin{array}{l}{H}_{rand}-{r}_{1}.\left|{H}_{rand}-2{r}_{2}.{H}_{t}\right| if {q}_{1}\ge 0.5 \\ {H}_{prey}-{H}_{mean}-{r}_{3}\left(LB+{r}_{4}\left(UB-LB\right)\right) if {q}_{1}\le 0.5\end{array}\right..$$

The characteristics and values of the parameters listed in the above equation are presented in Table 2. Prey escape energy is a fundamental component described as follows:

32 $$R=2{R}_{0}(1-\frac{t}{T}),$$

where *R* is the escape energy, ${R}_{0}$ is the initial energy, *J* is the prey force for random jumps. According to *J* and *R* parameters, the mathematical considerations related to the SS phase is be rewritten as follows:

**Table 2:** The parameters characteristics and values of the HHO algorithm.

Parameter	Symbol	Value
Random number	${q}_{1}$	[0, 1]
Next position of the Harris hawk	${H}_{t}+1$	–
Random position of the hunter	${H}_{rand}$	–
Current position of the hawk	${H}_{t}$	–
Average position of all falcons	${H}_{mean}$	–
Hunting position	${H}_{prey}$	–
Number of iterations	*t*	50
Random numbers	${r}_{1}..,{r}_{4}$	[0, 1]
Maximum number of iterations	*T*	55
Lowest bound	*LB*	–
Highest bound	*UB*	–
Escape energy	*R*	[− 2, 2]
Initial energy	${R}_{0}$	[− 1, 1]
Prey force for random jumps	*J*	–

33 $${H}_{t}+1= {H}_{prey}-{H}_{t}-R \left|J{H}_{prey}-{H}_{t}\right|,$$

34 $${J=2(1-r}_{5}), 0 <{r}_{5}<1.$$

The equation resulting from the HS will also be obtained based on the above explanations:

35 $${H}_{t}+1= {H}_{prey}-R \left|{H}_{prey}-{H}_{t}\right|$$

**Figure 8:**
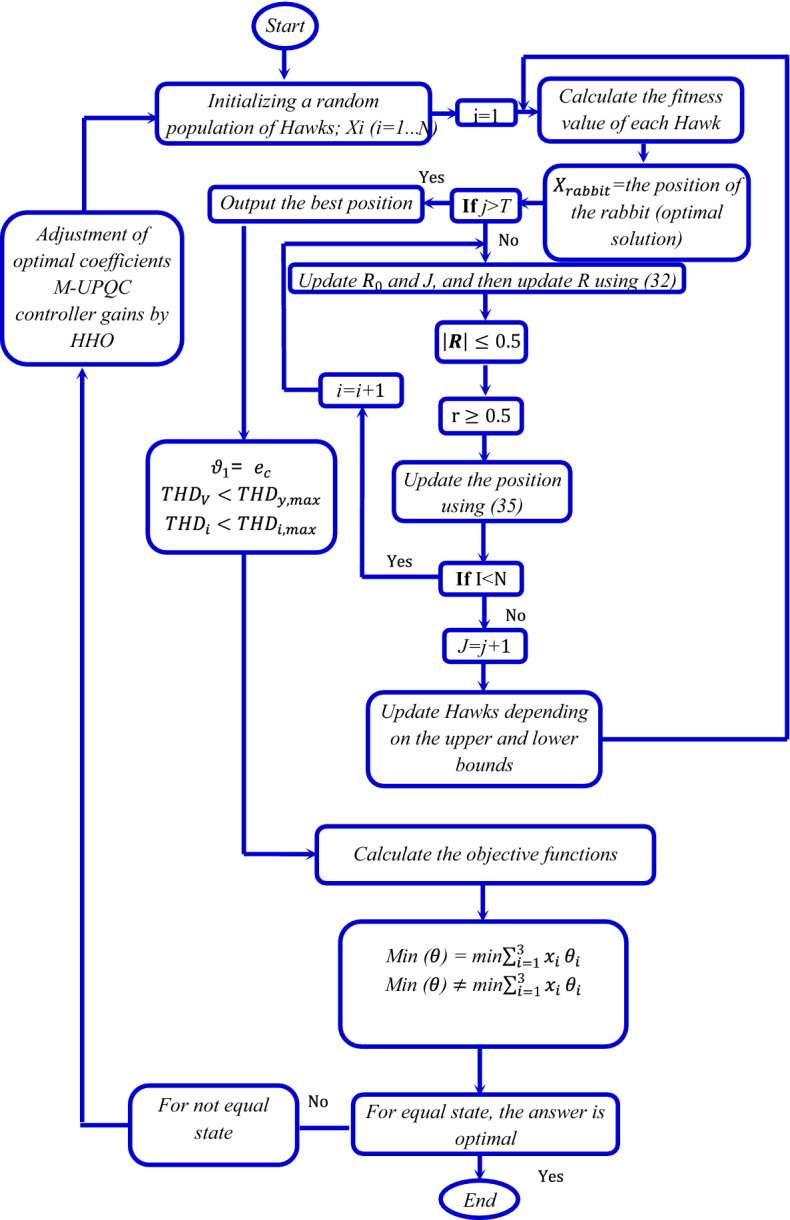
Flowchart of the proposed technique based on HHO method.

If at this stage the values are r $\ge 0.5$ and $\left|\mathrm{R}\right|\le 0.5$, it means that the prey is tired and the possibility of a surprise attack by the hawk is possible. The hierarchical process and implementation steps of defined OF in the HHO procedure is presented in Fig. 8.

### GOA method

The GOA procedure consists of two steps containing exploration and exploitation issues. The group manners of the grasshopper are modeled as follows:

36 $${\mathrm{X}}_{\mathrm{i}}={\mathrm{S}}_{\mathrm{i}}+{\mathrm{G}}_{\mathrm{i}}+{\mathrm{W}}_{\mathrm{i}},$$

where ${\mathrm{X}}_{\mathrm{i}}$ is the position of the ${i}^{th}$ grasshopper, ${\mathrm{S}}_{\mathrm{i}}$ is social interaction, ${\mathrm{G}}_{\mathrm{i}}$ is the gravity force on the ${i}^{th}$ grasshopper and ${\mathrm{W}}_{\mathrm{i}}$ is the horizontal force of the wind, by assuming random behavior, Eq. (36) will be rewritten as follows:

37 $${\mathrm{X}}_{i}={{r}_{a}S}_{i}+{{r}_{b}G}_{i}+{{r}_{c}W}_{i},$$

where ${r}_{a}, {r}_{b}$ and ${r}_{c}$ are random values [0, 1], respectively. If considered to be in the formation of a vector, relation (39) would define this vector.

38 $${d}_{ij}= \left|{x}_{j}-{x}_{i}\right|,$$

39 $$\widehat{{d}_{ij}}= \frac{\left|{x}_{j}-{x}_{i}\right|}{{d}_{ij}}.$$

**Figure 9:**
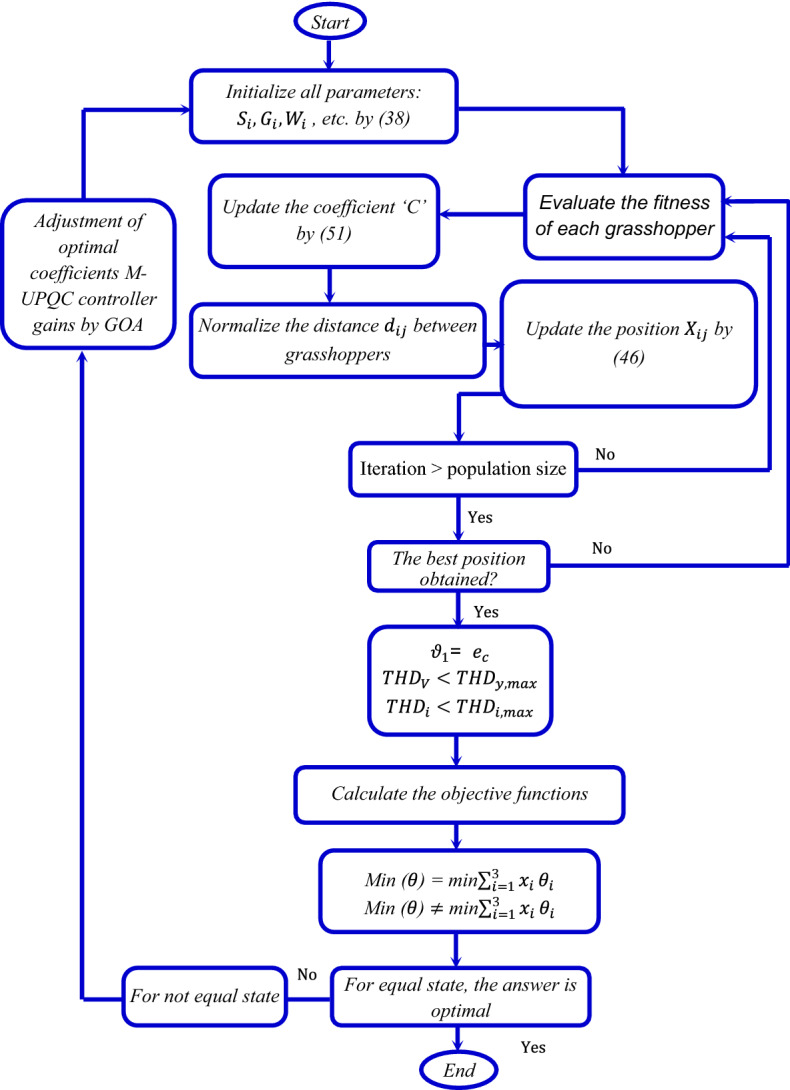
Flowchart of the proposed technique based on GOA method.

Social relations to be described in the form of a process:

40 $${P}_{(r)}=f{e}^{\frac{-r}{l}}-{e}^{-r},$$

where ‘*f’* represents the intensity of attraction and ‘*l’* represents the length scale. The revamped components of gravity and horizontal wind power will be developed as follows:

41 $${G}_{i}= -g\widehat{{e}_{g}},$$

42 $${W}_{i}= u\widehat{{e}_{w}},$$

where ‘*g’* is the gravitational constant, $\widehat{{e}_{g}}$ is the unit vector toward the center of the earth, *u* is the constant thrust, and $\widehat{{e}_{w}}$ is the unit vector in the direction of the wind. Based on the cited issues, relation (37) can be rewritten as follows:

43 $${X}_{i}^{d}=c\left[\sum_{\begin{array}{c}j=1\\ j\ne i\end{array}}^{N}c\frac{{ub}_{d}-{lb}_{d}}{2}s\left(\left|{x}_{j}^{d}-{x}_{i}^{d}\right|\right)\frac{{x}_{j}-{x}_{i}}{{d}_{ij}}\right]+\widehat{{T}_{d}},$$

where ${ub}_{d}$ is the upper bound in the ${D}^{th}$ dimension, ${lb}_{d}$ is the lower bound in the ${D}^{th}$ dimension ${P}_{(r)}$=*f *${e}^{\frac{-r}{l}}$*-*${e}^{-r}$ (40), $\widehat{{T}_{d}}$ is the value of the ${D}^{th}$ dimension in the target, and *c* is a decreasing coefficient to shrink the comfort zone, repulsion zone, and attraction zone. Further, the ‘C’ component must be decreased in ratio to the number of iterations to counterbalance exploration and exploitation levels^38^. The coefficient ‘C’ lowers the amenity zone on appropriateness to the number of interactions, and is estimated as follows:

44 $$C={C}_{max}-l\frac{{C}_{max}-{C}_{min}}{L},$$

where ${C}_{max}$ and ${C}_{min}$ represent the maximum and minimum values, ‘*l’* represents the current interaction and ‘*L’* represent the maximum number of interactions. The hierarchical process and implementation steps of defined ‘MOFs’ in the GOA procedure is presented in Fig. 9.

## Results and discussion

Here, the results and discussion are achieved by two methods, considered simulation and experimental analysis, further, which will be presented separately.

### Simulation results

**Figure 10:**
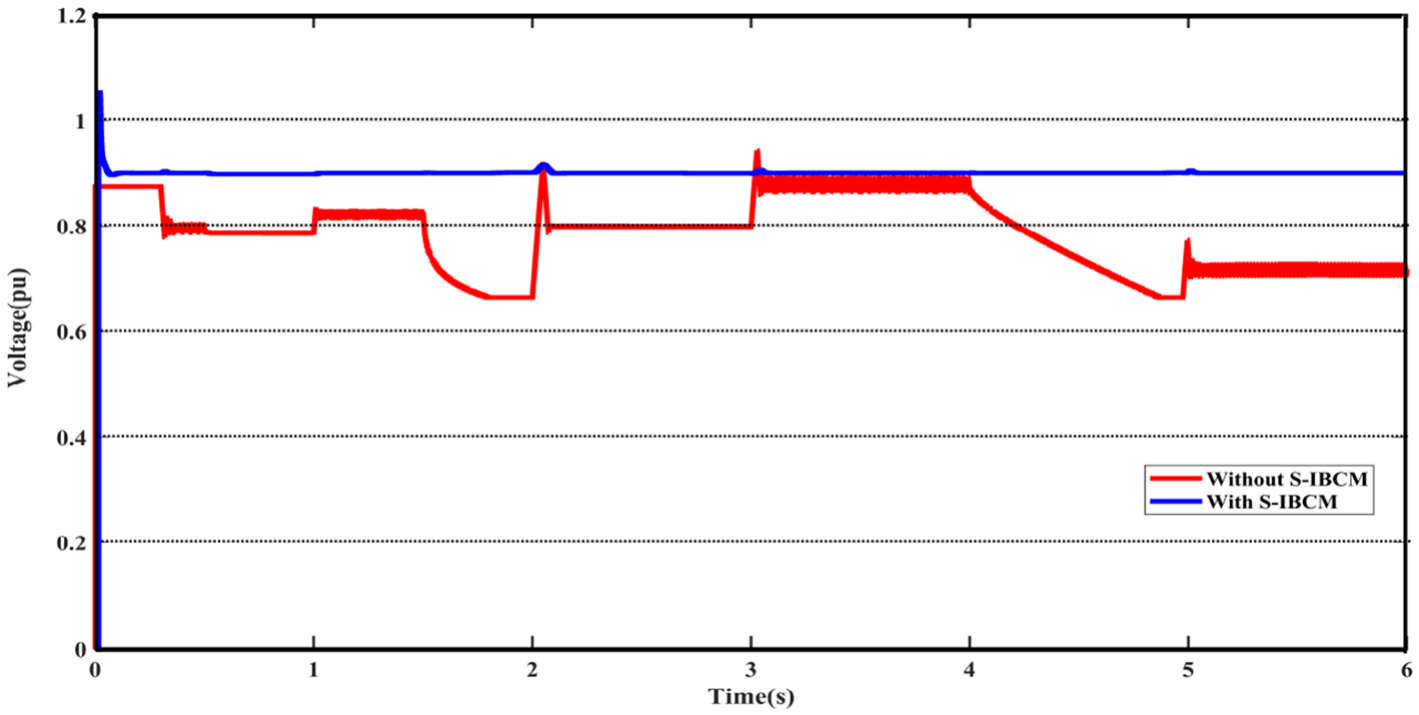
The DCMG terminal voltage with and without S-IBCM.

Based on the content of the study, the simulation results consist of two phases. The first phase includes investigating the impact of the S-IBCM component on the PV and BESS systems in the DCMG part. The second phase contains the DERs' operation and focuses on their performance with the presence of the M-UPQC module in the ACMG section. In the first phase, the scenario includes the output of the voltage and current parameters of the DC network, further will be discussed. About the first phase, Fig. 10 shows the system voltage at the DC grid output terminal (DCMG). DCMG output voltage values with and without the presence of the S-IBCM in the form of a plot presented in Fig. 10. By examining the output without S-IBCM, it is clear that the voltage parameter has strong fluctuations in several working points due to the operating conditions. The range of mentioned fluctuations reaches up to 0.35 pu.

**Figure 11:**
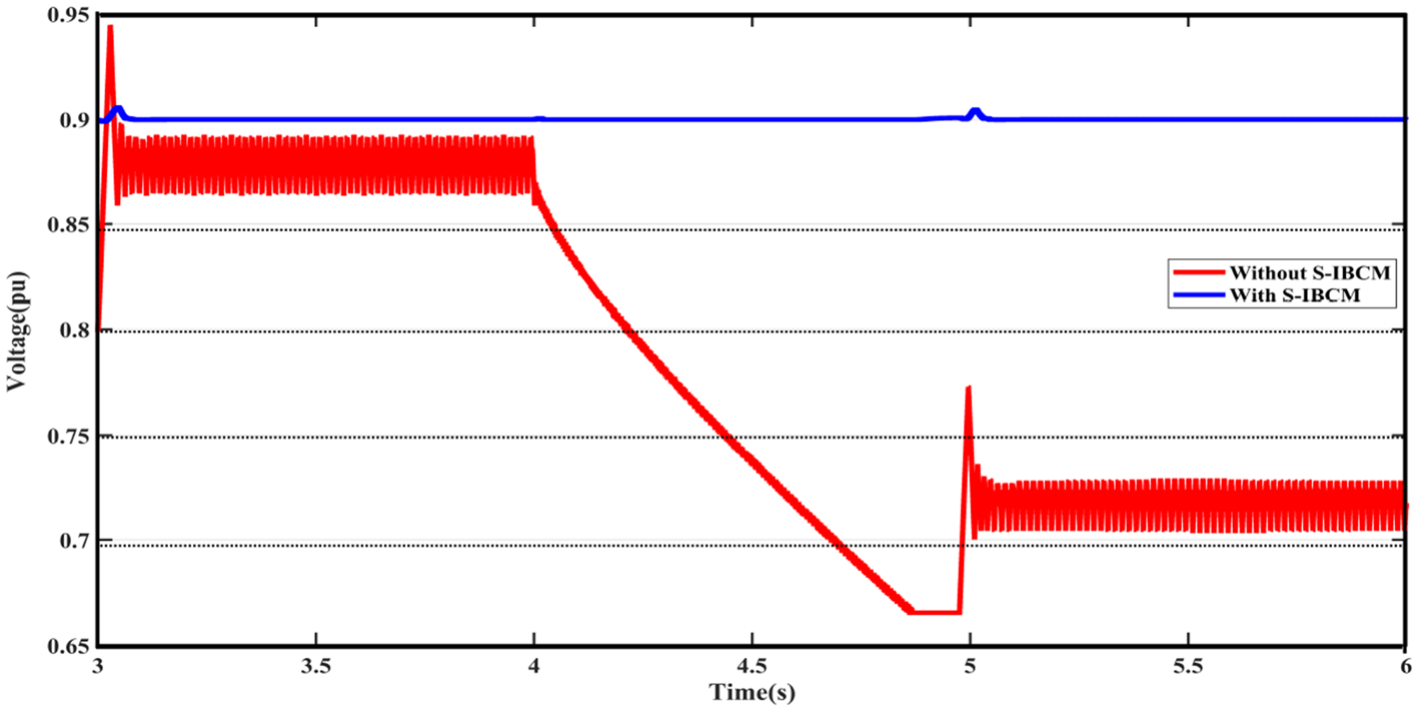
The open view of the DCMG terminal voltage with and without S-IBCM.

**Figure 12:**
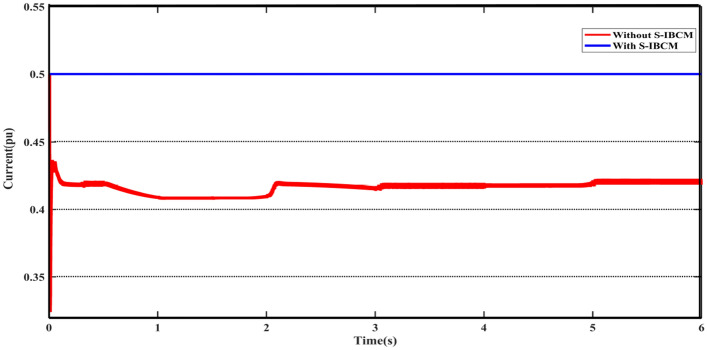
The DCMG terminal current with and without S-IBCM.

**Figure 13:**
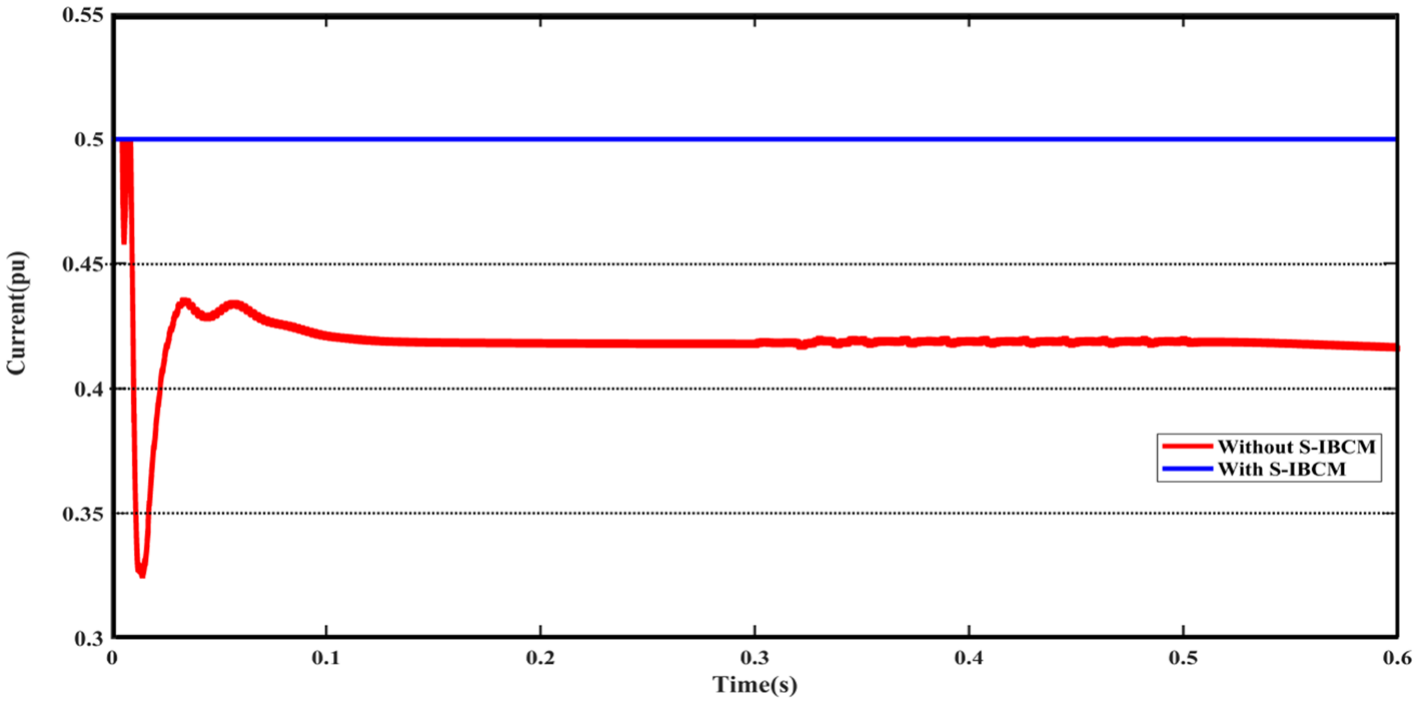
The open view of the DCMG terminal voltage with and without S-IBCM.

According to the settings and limitations defined for S-IBCM, the system voltage parameter reaches fixed at 0.9 pu. To S-IBCM influence, the broadened view of DCMG voltage output has been presented in Fig. 11. The mentioned plot shows the end section (3 s) of the DCMG output and describes the unsuitable operating conditions of the system without the presence of S-IBCM. Figure 12 shows the system flow parameter in the DCMG section. This output is similar to the system voltage parameter, it will be associated with fluctuation in several points. By S-IBCM operating, furthermore to the smoothness of the current output waveform, the output value is fixed to 0.5 pu. Figure 13 shows the DCMG current section at the initial moment. The absence of an initial drop in commissioning is one of the S-IBCM indicators. At the end of this phase, it cannot ignore the S-IBCM’s significant effect in eliminating fluctuations and increasing parameters (voltage and current) output values. Therefore, each of the mentioned parameters (V and I) has an increase of 20% and 15%, respectively.

**Figure 14:**
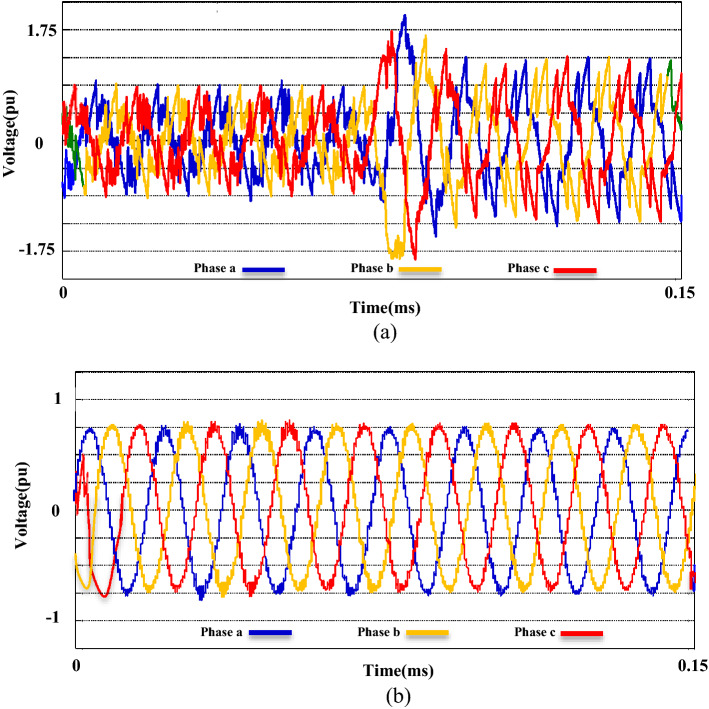
Three phase voltage extreme disturbances; (**a**) without the M-UPQC module, (**b**) with the M-UPQC module.

**Figure 15:**
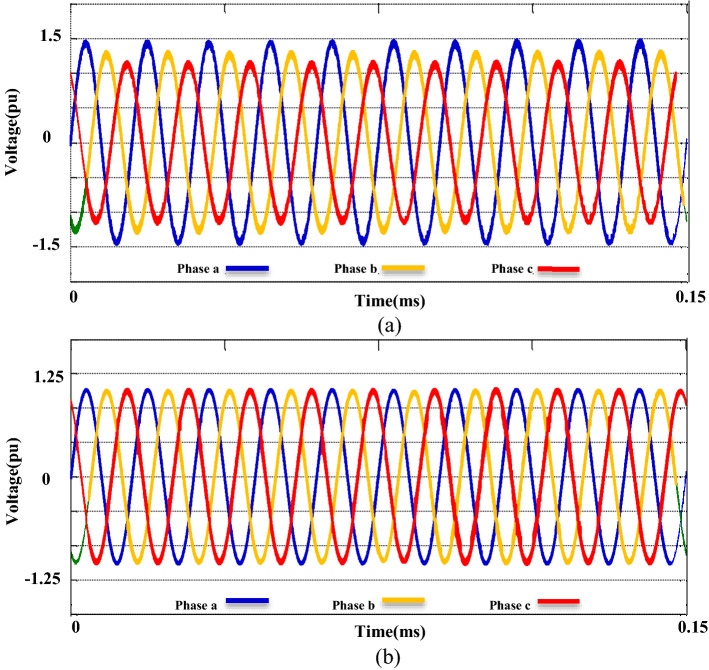
Three phase unbalanced output voltage; (**a**) without the M-UPQC module, (**b**) with the M-UPQC module.

**Figure 16:**
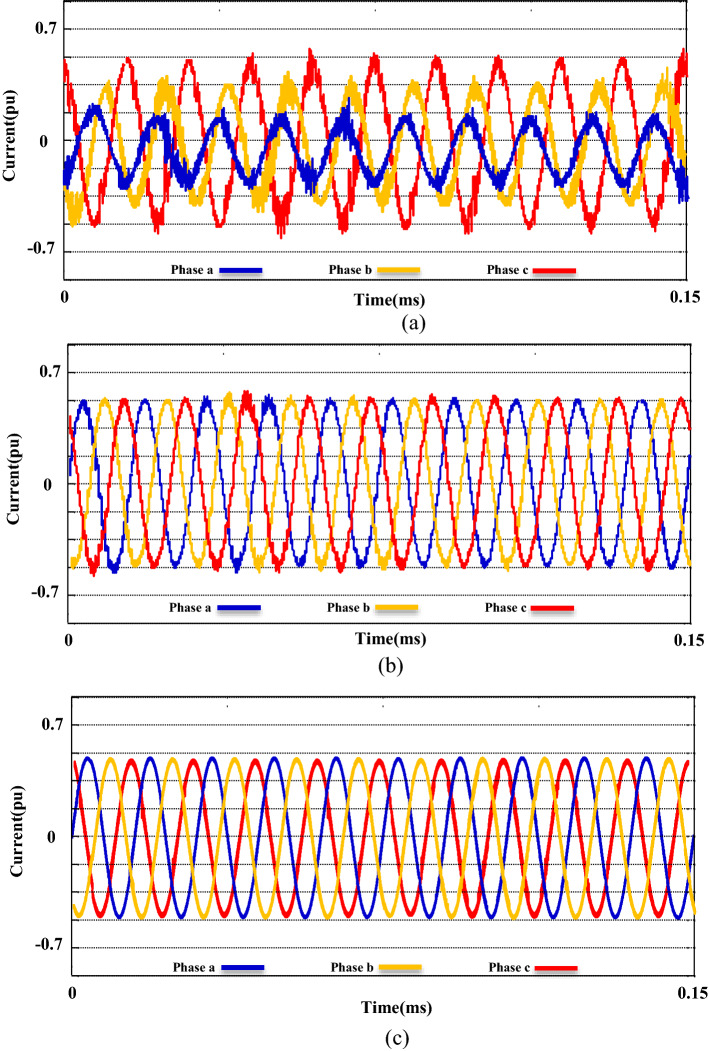
The current THD of the system: (**a**) Without compensator devices. (**b**) With the UPQC module. (**c**) With the M-UPQC module.

**Figure 17:**
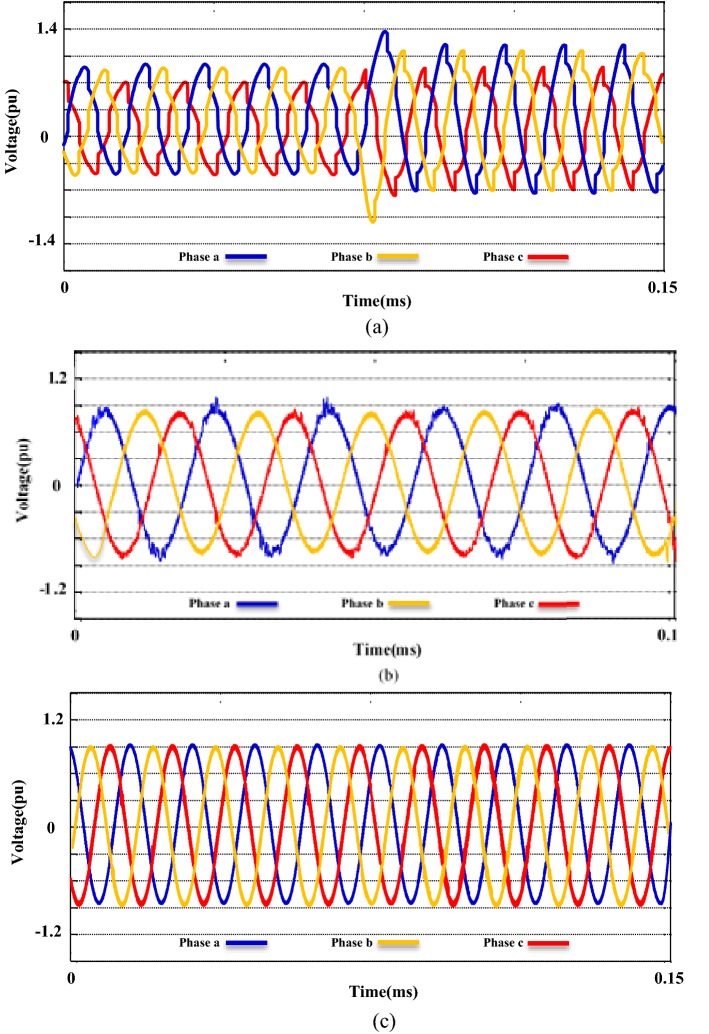
The voltage THD of the system; (**a**) without compensator devices, (**b**) with the UPQC module, (**c**) with the M-UPQC module.

The second phase of the simulations is exclusively related to the ACMG part. In this section, the fundamental parameters' behavior of the system is considered with and without the presence of compensators. The main topic of this section includes ‘PQ’ issues required of consumers in the ACMG corridor. The ‘PQ’ indicates fluctuations, unbalance, and harmonics. To analyze the behavior of the M-UPQC module, the traditional UPQC is considered a complementary device. Figure 14 shows the three-phase voltage extreme disturbances of the ACMG operation. In the first part (a), the system voltage does not have favorable conditions and contains many disorders due to the connection to non-linear loads. The second part (b) shows the system voltage with the duplicate operating conditions as before (supplying non-linear loads) with the presence of the M-UPQC module. The voltage waveform output is significantly improved, although the harmonic issue of the waveforms is discernible. The issue of network voltage imbalance is another factor presented in Fig. 15. In the first part (a), the system voltage waveform has unbalanced with a variable amplitude of 1.5 pu (without the preferred module). In the second part (b), by M-UPQC, the system unbalanced be improved to an acceptable level in the 1 pu value. The next item in the ‘PQ’ subject is harmonics which is typically defined as a total harmonic distortion (THD) component in electrical standards. In this study, the mentioned components for two parameters (voltage and current) has presented with symbols ${THD}_{v}$ and ${THD}_{i}$. Here, the components' behavior in the ACMG section will be explained with and without UPQC and M-UPQC equipment, respectively. Figure 16 shows the desired network current THD (${THD}_{i}$) displayed in the three separate plots structure. Based on the output obtained in 16(a), the system output current has a large amount of oscillation in addition to harmonics, which recorded ${THD}_{i}$ 28.1% value. In the following, by using UPQC, the problem of unbalanced waveforms is almost improved, and the recorded value of the threat component (18.5%) shows the adequate performance of UPQC. In the third part (c), after operating M-UPQC, the working conditions of the system current improve significantly, so the recorded ${THD}_{i}$ value (5%) is in the acceptable range (2%-5%). Figure 17 shows the desired network voltage THD (${THD}_{v}$) displayed in the three separate plots structure. According to the waveforms presented in part (a) of the mentioned figure, unlike the system current, the network voltage component in the state without operating compensating devices has far better conditions. Based on the output obtained in 17(a), the system output voltage has a large amount of oscillation in addition to harmonics, which recorded ${THD}_{v}$ 23.2% value. In the following, by using UPQC, the problem of unbalanced waveforms is almost improved, and the recorded value of the threat component (13.3%) shows the adequate performance of UPQC. In the third part (c), with the execution of the hierarchical suggested control embedded in the M-UPQC equipment, the ${THD}_{v}$ value has decreased to the allowed range and will stabilize at 3.5%. The optimized control coefficients by the applied optimization algorithms are indicated in Table 3. The harmonic component results (${THD}_{v}$ and ${THD}_{i}$) are shown in Table 4.

**Table 3:** Optimized PI controller gains of PI-MR and resonant gain factor.

Procedure	${Kp}_{i}$			${Ki}_{i}$			
HHO	2.319			4.537			
GOA	1.688			3.355			
BHO	0.939			1.445			
Resonant gain	${K}_{1}$	${ K}_{3}$	${ K}_{5}$	${K}_{7}$	${K}_{9}$	${ K}_{11}$	${ K}_{13}$
Value of *K* factor	11.2	9.5	8.4	7.7	7.1	5.8	4.3

**Table 4:** The harmonic output voltage and current values.

Parameters	Without compensator	With UPQC	With M-UPQC
THDv (%)	23.2	13.3	3.5
THDi (%)	28.1	18.5	5

### Experimental results

**Figure 18:**
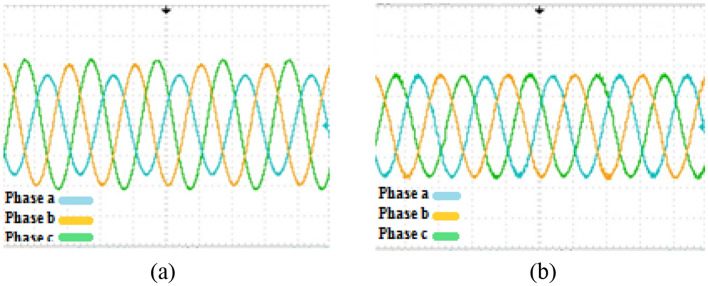
The experimental results of the voltage unbalance; (**a**) without compensator, (**b**) with M-UPQC.

**Figure 19:**
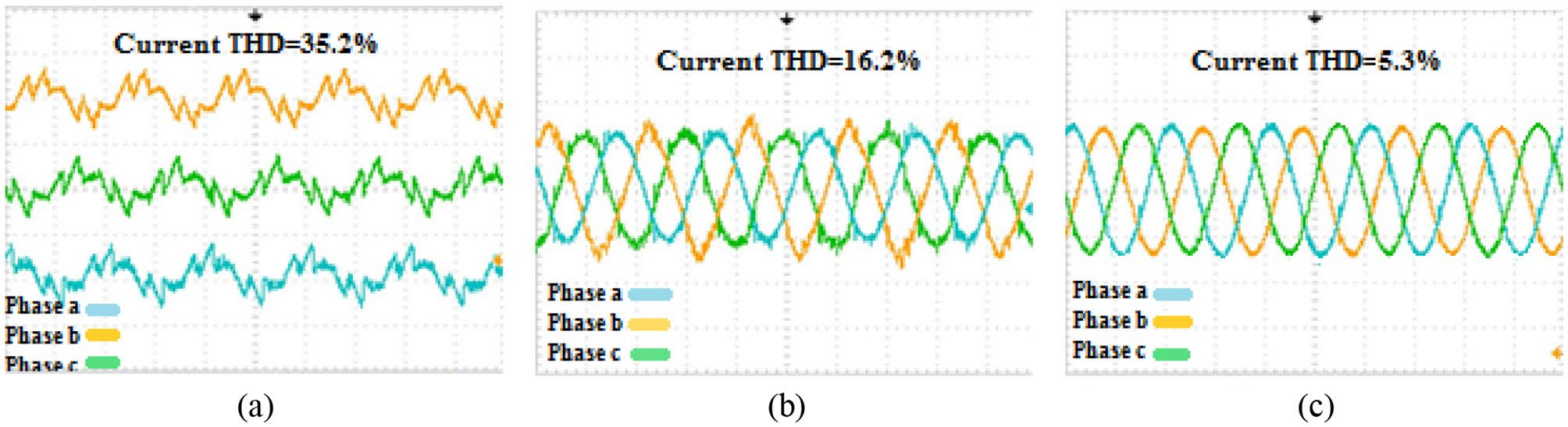
The experimental results of the current THD; (**a**) *THDi* without compensator, (**b**) *THDi* with UPQC, (**c**) *THDi* with M-UPQC.

**Figure 20:**
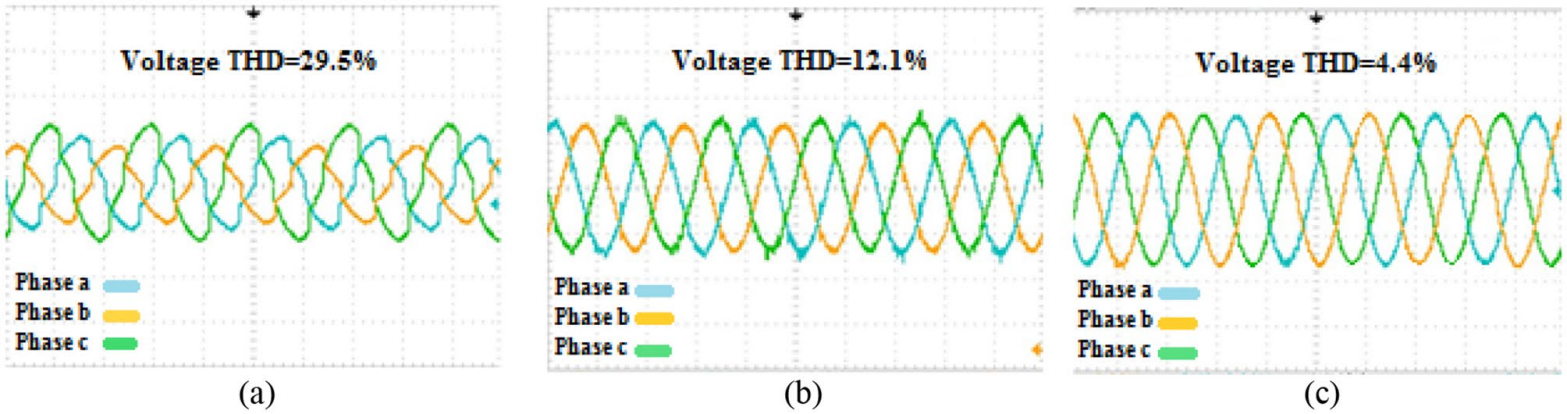
The experimental results of the voltage THD; (**a**) *THDv* without compensator, (**b**) *THDv* with UPQC, (**c**) *THDv* with M-UPQC.

**Figure 21:**
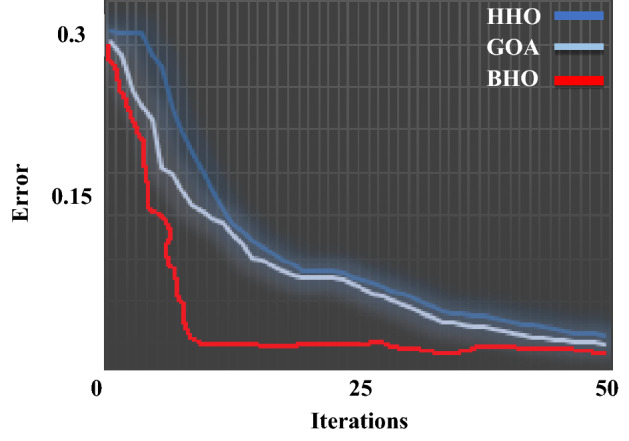
The iteration diagram of algorithms.

The productiveness of the suggested M-UPQC is confirmed by experimental analyses carried out on a DSP processor in the lab-scale prototype. All technical specifications of the laboratory platform are provided in the appendix. The results achieved from the configuration of the proposed M-UPQC in the real-time state have been presented in Figs. 18, 19 and 20. The mentioned outputs include three PQ components and contain voltage unbalance, ${THD}_{i}$, and ${THD}_{v}$, respectively. Figure 18 consists of two parts, the first part (a) shows the operation of the case study without M-UPQC, in the following, the voltage unbalanced is quite evident. In the second part (b), by the compensator (M-UPQC), the mentioned problem has been completely solved. The amplitude of voltage unbalanced is approximately 25%. Figure 19a–c shows the ${THD}_{i}$ of the system with and without the proposed apparatuses. The ${THD}_{i}$ values are revamped from 35.2 to 16.2% by the UPQC module, furthermore, the cited perturbations have decreased to 5.3% by the embedded proposed technique in the M-UPQC controller. Figure 20a–c shows the measured values of ${THD}_{v}$ with and without the proposed apparatuses. The intensity of harmonic disturbances in ${THD}_{v}$ is a little lower than ${THD}_{i}$, and this issue indicates the suitable conditions for relatively the system voltage better operation. According to the acquired results, the ${THD}_{v}$ quantity will be decreased from 29.5 to 12.1% by UPQC compensating component, and then by M-UPQC, the ${THD}_{v}$ will be damped at 4.4%. The measured values of harmonic components of voltage and current in the lab-scale prototype are presented in Table 5. The experimentally achieved results confirm the system disturbances amount and rate (THD) resulting from the technique under the study software platform. Furthermore, the simulation output results show the comparative superiority of the implementation and efficiency of the proposed control technique for the M-UPQC system compared to the conventional UPQC. Among the presented techniques, the best performance is related to the BHO approach with high convergence velocity. Finally, GOA and HHO algorithms are placed in the subsequent ranks, respectively. The mentioned matter is verified by the iteration diagram for each algorithm as shown in Fig. 21.

**Table 5:** The harmonic output voltage and current values.

Parameters	Without compensator	With UPQC	With M-UPQC
THDv (%)	29.5	12.1	4.4
THDi (%)	35.2	16.2	5.3

Here, the implementation and installation of proposed compensators are usually more economical for large-scale systems. Furthermore, the proposed modules can be considered based on other control methods such as predictive control, and fuzzy control for forthcoming studies and research. Moreover, another topic that can be impressive in the proposed method development in this paper is to improve the control response level of modules by operating algorithms with a high convergence velocity.

## Conclusion

This study aimed to improve the PQ operation for an HMG. For this purpose, two scenarios have been considered for the grid study separately. The first scenario includes the implementation of the S-IBCM interface in DCMG as a complementary module to increase PV and BESS output efficiency. The second scenario is the execution of M-UPQC on the ACMG to enhance PQ indicators. Here, the first challenge is to adjust embedded paths in the S-IBCM equipment. Furthermore, the next challenge is to modify the M-UPQC controller parameters (PI-MR) by the BHO, HHO, and GOA techniques. The first scenario results show that the S-IBCM interface effectiveness in the DCMG terminal voltage and current includes a significant reduction in the fluctuation range of the mentioned components and increased the output of these parameters to 20% and 15%, respectively. Based on the second scenario, the technique applied to the M-UPQC module remarkably enhanced power quality parameters such as voltage oscillation, voltage unbalance, and harmonic components (${THD}_{v}$ and ${THD}_{i}$) of the ACMG compared to the conventional type (UPQC). In this paper, the BHO approach was the best performance compared to the HHO and GOA algorithms. Further, the experimental outputs confirm the results obtained from the case study simulation. Future research can address power quality components of the AC/DC MG systems concerning suitable productivity and increasing the growth of these grids.

## Supplementary Information

Supplementary Information.

## Data Availability

The datasets used and/or analyzed during the current study available from the corresponding author on reasonable request.
