# Supplementary material for: Improvement of power quality parameters using modulated-unified power quality conditioner and switched-inductor boost converter by the optimization techniques for a hybrid AC/DC microgrid
Source: Sci Rep. 2022 Dec 15;12:21675. doi: 10.1038/s41598-022-26001-8 (PMC9755313; doi:10.1038/s41598-022-26001-8)
Supplement: Supplementary file 1 — Supplementary Information. [file 41598_2022_26001_MOESM1_ESM.pdf]

**M-UPQC specification**
L_{sd} = 1.95 mH, $R_{L_{csc}}$= 0.35 Ω, L_{pd} = 1.25 mH, $R_{L_{vsc}}$ = 0.3 Ω, C_{dc} = 1860 μF
**ACMG specification**
The loads of the ACMG system are including linear load (1.3 MVA with 0.85 PF), and nonlinear load (0.95 MVA with 0.8 PF). The nominal power for each equipment such as MTGs, DFIG, respectively: 15×5.5 kW, 14×180 kW
**Laboratory platform specification**
DSP model: dSPACE 1104, Sampling time: 30μs, Switching frequency: 35 kHz, Fundamental frequency: 50Hz, Grid voltage: 110V, Source inductance: 33mH, Nonlinear load:100Ω/45mH

Table S1: Grid, module and laboratory platform specifications
